# Blurring Boundaries: The Overlap of Autistic Traits and Emerging Psychotic Symptoms

**DOI:** 10.1192/j.eurpsy.2026.12158

**Published:** 2026-07-31

**Authors:** G. Lacerda, M. Soares, I. G. Mata, M. Cunha

**Affiliations:** 1 Unidade Local de Saúde da Arrábida, Setúbal; 2 Unidade Local de Saúde Arco Ribeirinho, Barreiro; 3 Unidade Local de Saúde Loures-Odivelas , Loures, Portugal

## Abstract

**Introduction:**

The identification of psychotic symptoms in individuals with Autism Spectrum Disorder (ASD) poses a substantial clinical challenge. Symptom overlap between ASD and psychosis, combined with diagnostic overshadowing, where comorbidities are incorrectly attributed to autism, complicates early detection and intervention. Research has increasingly focused on the nuances of differentiating these conditions to improve clinical outcomes.

**Objectives:**

This narrative review aims to (1) explore the difficulties in recognizing psychotic symptoms in individuals with ASD, (2) examine factors contributing to diagnostic overshadowing, and (3) summarize current recommendations for improving differential diagnosis and clinical assessment.

**Methods:**

Evidence-based review, through research conducted on PubMed and selection of the most relevant studies on this topic, published in the last decade.

**Results:**

Findings indicate that social deficits, restrictive behavioral patterns, and unusual beliefs characteristic of ASD can mimic or mask psychotic symptoms. Traditional psychosis risk assessment tools often have reduced sensitivity in autistic populations, which may delay recognition and treatment. Diagnostic overshadowing remains a persistent barrier, emphasizing the need for clinician awareness and specialized training. Literature highlights the importance of integrating multiple assessment strategies, including clinical interviews adapted for ASD, caregiver input, and longitudinal monitoring, to improve diagnostic accuracy. Communication deficits, alexithymia, and reduced insight complicate self-report reliability, while clinicians’ biases may lead to both over- and under-diagnosis of psychosis in ASD.

**Conclusions:**

Accurate identification of psychotic symptoms in individuals with ASD requires a nuanced understanding of overlapping behaviors, specialized assessment tools, and increased clinical vigilance. Tailoring evaluation strategies and training clinicians can facilitate differential diagnosis, prevent treatment delays, and improve patient outcomes.

**Disclosure of Interest:**

None Declared

